# A carcinoid tumor arising from a mature cystic teratoma in a 25-year-old patient: a case study

**DOI:** 10.1186/s12957-016-0867-8

**Published:** 2016-04-21

**Authors:** Joo-Yeon Kim

**Affiliations:** Department of Pathology, Haeundae Paik Hospital, University of Inje College of Medicine, 1435 Jwa-dong, Haeundae-gu Busan, Republic of Korea

**Keywords:** Ovary, Teratoma, Carcinoid tumor

## Abstract

**Background:**

Mature cystic teratomas (MCTs) are common benign tumors occurring in the ovaries. Malignant transformation of teratomas (TMT) occurs in 1–3 % of all MCTs, usually in postmenopausal women. Squamous cell carcinoma is the most common tumor type. Primary carcinoid tumors of the ovary are uncommon, representing only 0.3 % of all carcinoid tumors and less than 0.1 % of all ovarian cancers.

**Case presentation:**

A carcinoid tumor of the trabecular type arising from a MCT is presented in a 25-year-old woman. TMT was detected incidentally. Histologically, uniform, polygonal cells were arranged in a cord and trabecular pattern. Immunohistochemical staining showed positivity for neuro-specific enolase, synaptophysin, and CD56.

**Conclusions:**

This case represents a very rare example of carcinoid tumor occurring in a woman younger than 30 years of age. Our findings suggest that sufficient tumor sampling is necessary to avoid overlooking small lesions, which, in our case, were not detected on either radiologic or gross examination.

## Background

Malignant transformation of teratoma (TMT) is uncommon condition occurring in 1–3 % of all mature cystic teratomas (MCTs) [1]. Moreover, TMT tends to occur in postmenopausal women, especially those who are 15 years older than conventional MCT [2]. The most common malignant tumor type in MCTs is squamous cell carcinoma, followed by adenocarcinoma and carcinoid tumors. Carcinoid tumors of the ovary are uncommon, particularly primary carcinoid tumors, representing only approximately 0.3 % of all such tumors [3].

Herein, we report a rare case of carcinoid tumor arising from a MCT in a 25-year-old nulliparous woman.

## Case presentation

A 25-year-old woman (gravida 0, para 0) presented with a 4-year history of an asymptomatic pelvic mass that had increased in size in the previous few months. The women had an unremarkable medical and gynecological history with a regular menstrual cycle.

**Fig. 1:**
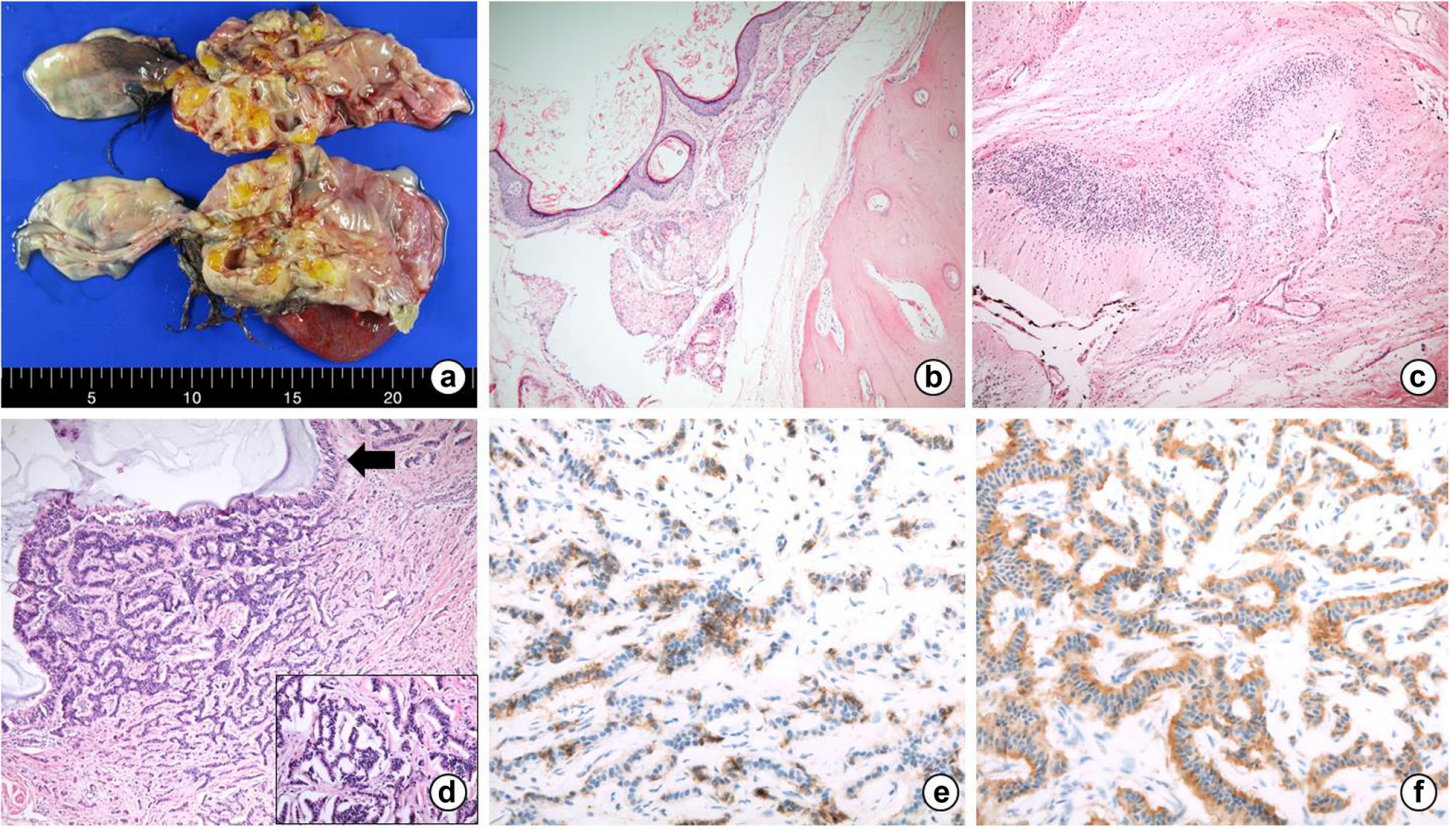
**a** Gross appearance of the left ovary: A multicystic mass containing viscous sebaceous material and hair is visible. **b**, **c** Uniform cells are arranged in cords and trabeculae beneath the respiratory epithelium. **d**, **e** Tumor cells showing positivity for CD56 and synaptophysin. **f** The Ki-67 proliferative index in the carcinoid tumor is 1 %

Ultrasonography confirmed the presence of a hypoechoic cystic mass of undetermined origin. Further evaluation was performed, including laboratory tests and computed tomography (CT). The serum level of CA 125 was found to be elevated (139.0 U/mL), and a CT scan revealed tumors in both the ovaries. The right ovary showed a 7.0 × 4.5 cm fat-containing cystic mass with internal calcification, and the left ovary showed 16.0 × 14.0 cm fat-containing cystic mass with multiple septa and internal calcification (Fig. 1a). The radiologic findings suggested that both the ovarian tumors were mature teratomas, and the patient underwent bilateral ovarian cystectomy. Macroscopically, the left ovary revealed a multicystic mass containing viscous sebaceous material and hairs, measuring 20.0 × 12.0 × 7.5 cm. The cystic wall also contained adipose and bony tissue. Similarly, the right ovary showed a cystic mass filled with sebum and hairs, measuring 6.5 × 5.0 × 4.0 cm. Frozen sections of the left ovarian mass were made. On microscopic examination, mature squamous and respiratory epithelium, as well as mesenchymal tissue including the bone, cartilage, smooth muscle, and adipose tissue were observed. In addition, abundant brain tissue, such as glial tissue and choroid plexus, were observed. Through the frozen sections, the left ovarian mass was diagnosed as MCT, and therefore, no further intervention was performed at the time of the operation.

However, formalin-fixed, paraffin-embedded multiple sections of the left ovary revealed an incidental microscopic focus that displayed a monomorphic population of polygonal cells beneath the respiratory epithelium. The focus measured 0.5 cm in diameter and was not observed in the frozen sections. The tumor cells formed cords and trabeculae and had scant, eosinophilic cytoplasm, and round nuclei with mild atypia (Fig. 1b, c). Immunohistochemical staining showed positivity for neuro-specific enolase (NSE), synaptophysin, and CD56 (Fig. 1d, e). One mitosis was seen per 10 high-power fields, and the Ki-67 proliferative index was 1 % (Fig. 1f). Therefore, the tumor was classified as low grade. Based on the morphological and immunohistochemical findings, the tumor was diagnosed as a carcinoid tumor (trabecular type) arising from a MCT. The right ovary, on the other hand, had no evidence of somatic type tumors.

The patient received no further treatment. And she had no clinical symptoms associated with carcinoid syndrome including cardiac involvement that might be a major cause of death in this syndrome, so cardiac ultrasound had not performed. Also, no evidence of recurrence or metastasis was detected on abdomen and pelvic computer tomography (CT) after 8 months of follow-up.

## Conclusions

MCTs account for approximately 20 % of all ovarian neoplasms, and are often diagnosed in young women [4]. Teratomas consist of diverse tissues derived from the three germ cell layers, the ectoderm, mesoderm, and endoderm. MCTs are usually benign; however, in 1–3 % of cases, they undergo TMT. Furthermore, patients with TMT are 15 years older than those with MCT without malignant transformation. The most common tumor type is squamous cell carcinoma, comprising about 80 % of cases of TMT. Other tumor types, including adenocarcinoma, thyroid carcinoma, and carcinoid tumor, have also been reported [1, 5].

Primary carcinoid tumors of the ovary are uncommon, accounting for only 0.3 % of all carcinoid tumors and less than 0.1 % of all ovarian cancers. Carcinoid tumors most commonly occur in the gastrointestinal tract. The second most common organ in which these tumors occur is the lungs. Ovarian carcinoid tumors may be primary or metastatic [3]. Most primary ovarian carcinoid tumors are unilateral; however, in 16 % of cases, the contralateral ovary displays a cystic teratoma or a mucinous neoplasm [6].

Primary ovarian carcinoid tumors are classified into four types, i.e., the insular, trabecular, mucinous, and mixed types. The most common type is the insular type [7]. Primary ovarian carcinoid tumors, in particular one third of the insular type, have been associated with the carcinoid syndrome despite the absence of metastasis [4]. The other types of primary ovarian carcinoid tumors are not usually associated with carcinoid syndrome.

In the present case, the patient had a trabecular type carcinoid tumor and did not have any symptoms of carcinoid syndrome for the previous 4 years.

Preoperative diagnosis of MCT of the ovary can be made through radiologic findings considering the presence of the fat tissue, hairs, bone, and cartilage within the tumor. However, TMT is not easily recognized. In most cases, a definitive diagnosis is possible only on postoperative examination of multiple tissue sections. Similarly, a small carcinoid tumor was identified incidentally in the present case. Tumor cells revealed immunoreactivity for the neuroendocrine markers following NSE, synaptophysin, and CD56, and these findings are typical of carcinoid tumors. This case suggests that, in teratomas, sufficient sampling from solid components is important not to miss the microscopic small areas showing TMT.

The optimal treatment strategy for TMT remains the main challenge. Surgical excision is regarded as the first approach [8]. A more aggressive surgical treatment may be considered according to the age of the patient. TMT usually occurs in postmenopausal women; therefore, radical surgery such as hysterectomy and bilateral salpingo-oophorectomy may be considered in such cases. However, in younger women, particularly those that are nulliparous, preservation of fertility is important. Despite the fact that there is no consensus regarding the optimal TMT treatment strategy for younger patients, local excision seems to be the most rational option [9]. According to the literature, the prognosis of organ-confined tumors is excellent for primary ovarian carcinoid tumors and the 10-year survival rates are approximately 100 %. However, in the advanced state, the 5-year survival rate decreases to 33 % [10].

In conclusion, a primary carcinoid tumor arising from a MCT is very rare in young patients, particularly those less than 30 years old, as demonstrated in the present case. Moreover, preoperative identification of TMT is not easy. Therefore, examination of multiple sections is necessary to detect even the smallest focus showing malignant transformation within solid tumor components.

## Consent

Written informed consent was obtained from the patients for publication of these case reports and any accompanying images. Copies of the written consent are available for review by the Editor-in-Chief of this journal.

## References

[1] Kim SM, Choi HS, Byun JS (2003). Mucinous adenocarcinoma and stromal carcinoid tumor arising in one mature cystic teratoma of the ovary with synchronous cervical cancer. J Obstet Gynaecol Res.

[2] Bojana D, Elizabeth DE, Anais M (2007). Growing teratoma syndrome of the ovary: review of literature and first report of a carcinoid tumor arising in a growing teratoma of the ovary. Am J Surg Pathol.

[3] Boyraz G, Selcuk I, Guner G, Usubutun A, Gunalp GS (2012). A primary insular type carcinoid tumor arising in a mature cystic teratoma of the ovary: a case report. J Clin Case Rep.

[4] Robert JK, Maria LC, Simon H, Robert HY. WHO Classification of tumors of female reproductive organs. 4th ed. Lyon: International Agency for Research on Cancer (IARC); 2013; 60–66.

[5] Stamp GW, McConnell EM (1983). Malignancy arising in cystic ovarian teratomas. A report of 24 cases. Br J Obestet Gynaecol.

[6] Robby SJ, Norris HJ, Scully RE (1975). Insular carcinoid primary in the ovary. A clinicopathologic analysis of 48 cases. Cancer.

[7] Taleraman A, Kurman RJ (2010). Blaustein’s pathology of the female genital tract.

[8] Roy S, Mandal S, Saroha V (2008). Primary carcinoid tumor of the ovary: a case report. Arch Gynecol Obstet.

[9] Stamatios P, Ioannis K, Chrysoula MS (2013). Mature ovarian teratoma with carcinoid tumor in a 28-year-old patient. Case Rep Obstet Gynecol.

[10] Kopf B, Rosti G, Lanzanova G, Marangolo M (2005). Locally advanced ovarian carcinoid. J Exp Clin Res.

